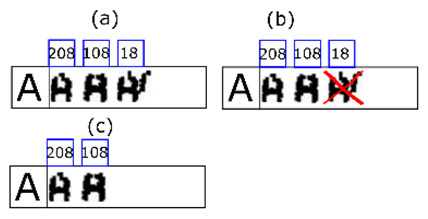# A Very High-Speed Validation Scheme Based on Template Matching for Segmented Character Expiration Codes on Beverage Cans †

**DOI:** 10.3390/s20113157

**Published:** 2020-06-02

**Authors:** José C. Rodríguez-Rodríguez, Gabriele S. de Blasio, Carmelo R. García, Alexis Quesada-Arencibia

**Affiliations:** Institute for Cybernetics, Campus de Tafira, Las Palmas de Gran Canaria, University of Las Palmas de Gran Canaria, 35017 Las Palmas, Spain; jcarlos@ciber.ulpgc.es (J.C.R.-R.); gabriel.deblasio@ulpgc.es (G.S.d.B.); ruben.garcia@ulpgc.es (C.R.G.)

**Keywords:** image processing, optical character recognition, OCR, pattern recognition, industrial inspection, very high-speed computing, character segmentation, tracking, template matching, supervised learning, unsupervised learning

## Abstract

This paper expands upon a previous publication and is the natural continuation of an earlier study which presented an industrial validator of expiration codes printed on aluminium or tin cans, called MONICOD. MONICOD is distinguished by its high operating speed, running at 200 frames per second and validating up to 35 cans per second. This paper adds further detail to this description by describing the final stage of the MONICOD industrial validator: the process of effectively validating the characters. In this process we compare the acquired shapes, segmented during the prior stages, with expected character shapes. To do this, we use a template matching scheme (here called “morphologies”) based on bitwise operations. Two learning algorithms for building the valid morphology databases are also presented. The results of the study presented here show that in the acquisition of 9885 frames containing 465 cans to be validated, there was only one false positive (0.21% of the total). Another notable feature is that it is at least 20% faster in validation time with error rates similar to those of classifiers such as support vector machines (SVM), radial base functions (RBF), multi-layer perceptron with backpropagation (MLP) and *k*-nearest neighbours (KNN).

## 1. Introduction

This paper expands upon a previous publication [1] and is the natural continuation of an earlier study [2] which presented an industrial validator of expiration codes printed on aluminium or tin cans, called MONICOD.

The literature on optical character recognition (OCR) is very extensive [3]. In fact, it has been a staple of computer vision since the 1950s. Great emphasis is usually placed on the quality of character recognition and the ability to generalise, which could be applied, for example, to the recognition of handwritten text. Relatively recently there has also been interest in trying to recognise other writing systems and alphabets [4].

Most of the work known to the authors is applicable to non-industrial environments [5,6]. Speed is often a secondary consideration. At best, it is intended to be manageable for humans, which is not the same as for industrial production.

Speed is precisely the main reason why MONICOD is a validator and not a recogniser [7,8]. In other words, it is a matter of verification, not recognition. With a validator the system knows what text is supposed to have been printed (the expected text) and its mission is to verify that the printed text is indeed legible and matches.

By contrast, with a recogniser, there is no expected code. The system does not know what text has been printed and its purpose is to try to identify it. The ability to generalise is very useful for this purpose. It is true that a recogniser can be used as a validator. If the printed text is readable by a human being, it must also be readable by an ideal recognition system. We may assume, therefore, that the text is unreadable if it is not recognisable. However, without an expected code, there is no point in asking whether the recognised text is “correct”. So there needs to be an expected text in order that it may be compared with the recognised text.

MONICOD, however, was designed as a validator, which has opened the door to important decisions that favour speed. Furthermore, as generalisation is not a critical aspect, the template matching method was chosen [9]. The existing literature for this kind of industrial validator has proven to be scarce [10,11,12,13]. Within this little explored field, with such singular demands, the authors present a solution with original proposals.

In this publication, the authors summarise in Section 2 and Section 3 the origin of the segmented shapes that will serve as system inputs. Section 4 and Section 5 will then explain the validation algorithm. For this validation algorithm to work, training is required, the details of which are referred to in Section 6. Section 7 and Section 8 provide a description of the experimental setup and results, which demonstrate the strengths and weaknesses of the proposed algorithm. Finally, Section 9 presents the conclusions and future work.

## 2. Character Segmentation Prior to Validation

The previous study [2] described a computer vision solution, MONICOD, which is an industrial validator of expiration codes printed on aluminium and tin cans. In this context “validate” means to determine whether the expiration code printed on the can is: (a) legible and (b) correct. In other words, a code is positively validated only if a human being can read it correctly without difficulty (a) and, in addition, the printed code corresponds to the expected code (b).

MONICOD’s absolute priority is to maximise the number of cans it is able to validate per unit of time with a minimum target of at least 35 cans per second. This requirement meets the specifications of the companies funding the research (Compañia Embotelladora de Canarias and Compañia Cervecera de Canarias), which seek to validate expiration codes on cans moving as fast as possible on the conveyor belt.

**Figure 1:**
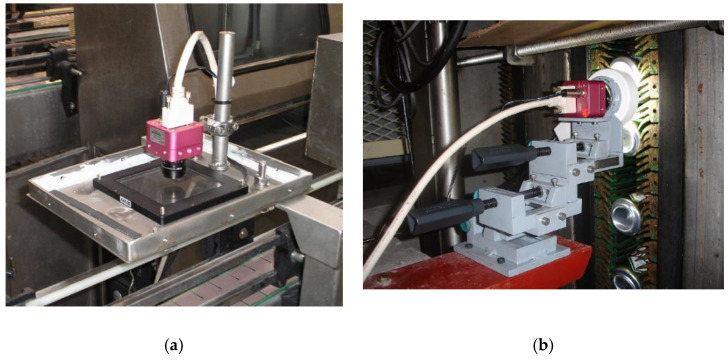
Image acquisition devices on a conveyor belt with a frame rate of 200 (black and white) images per second for (**a**) ground-plane line and (**b**) vertical plane.

MONICOD performs several processes arranged in sequence. First, images are recorded at a rate of 200 frames per second. A high-speed camera is used for this purpose. The images are captured in a controlled environment. To this end a camera obscura is used with a bright, homogeneous light source (see Figure 1). The cans are transported on the conveyor belt and pass under the camera obscura.

**Figure 2:**
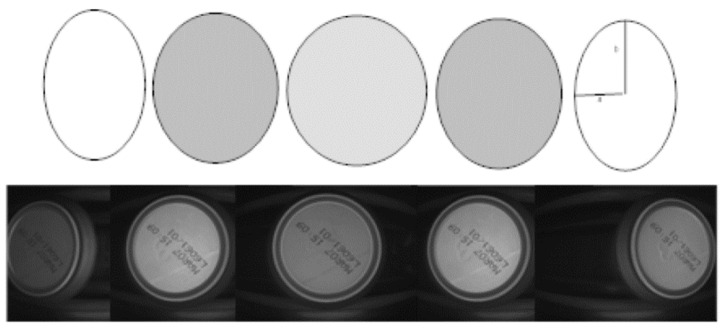
Selecting the best image for the can. The best image is the one in which the can is centred.

A frame rate of 200 images per second with cans passing through the field of view at a rate of 35 cans per second (maximum) provides several image captures of the same can. Consequently, the first process consists of selecting the best available image for that can. The best image (most suitable for an evaluation procedure) is that in which the can is most centred, minimising optical distortion (see Figure 2). The selection procedure also provides the area of interest of the selected image where the code should be found.

**Figure 3:**
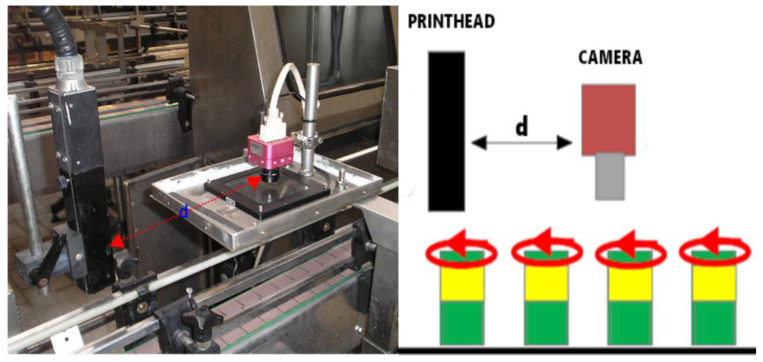
By reducing distance *d* (to less than one metre), the issue of rotational invariance is considerably mitigated.

It is interesting to note that the validation system presented in this article is NOT invariant under rotation. In order to address this issue, without added computational cost, a very simple hardware solution was chosen: the printing and image acquisition points were brought close enough together that the can does not have time to rotate appreciably between the two points (see Figure 3).

**Figure 4:**
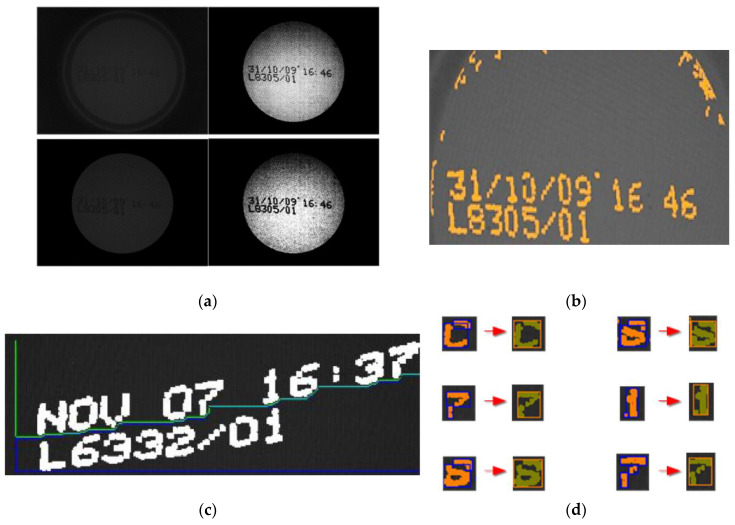
(**a**) Enhancement and equalisation of the area of interest. (**b**) Separation of ink and background. (**c**) Arranging the ink fragments into lines of text. (**d**) Grouping of ink fragments into characters.

Then, global enhancement and equalisation processes [14,15] are applied to the aforementioned area of interest to improve the visual quality of the area where the code is displayed. We call this stage “pre-processing” (see Figure 4a).

The next step is to extract and isolate the characters from the image. This is known as code segmentation [16]. To do this, the first phase consists of discriminating “ink-carrying” pixels from non-ink-carrying pixels [17,18,19] (see Figure 4b). Adjacent ink-carrying pixels are then grouped into fragments using a flooding technique. This starts with a pixel that has not yet been assigned to a fragment (a “seed”). From this seed the adjacent ink pixels are explored recursively (the implementation is in fact iterative). When no more adjacent ink pixels are located, all the discovered pixels are declared to be grouped in one fragment.

Each of these fragments is assigned to a “band” through the banding procedure (see Figure 4c). We use the term band to refer to the more or less horizontal strips where it is assumed that all the fragments within them correspond to text on the same line. Note that the expiration codes may be arranged on one or more lines. This novel procedure for assigning fragments to bands is described in [1]. It is based on agents that simultaneously navigate the image looking for line spacing in the segmented text. Fragments located between two consecutive line spacings are assigned to the same band.

At this point most of the fragments correspond to complete characters. However, there are a certain (low but significant) percentage of cases where the fragment is only a part of a character. For this reason, the grouping of fragments is evaluated in the last phase of segmentation (see Figure 4d). For this grouping to take place, a few fundamental rules must be followed. Fragments that can be grouped together must belong to the same band and a grouping is only feasible if it does not exceed a maximum width and height. Nor can it exceed a maximum number of pixels. Once these restrictions are overcome, it is the degree of overlap of the x-axis projections of the fragments under consideration that decides whether or not grouping takes place. The end result of this process is a collection of solitary fragments (which could not be grouped) and grouped fragments. This is what is known as a set of morphologies.

The last stage, which this paper deals with, consists of comparing these extracted morphologies with expected morphologies. All the expected morphologies correspond to characters, but this is not the case with the extracted morphologies: some may be noise that should be ignored by the system or character fragments that could not be successfully grouped. The test involves checking that all the expected characters are present and, moreover, that the characters are presented in the correct order.

## 3. Information Extracted in Previous Stages

**Figure 5:**
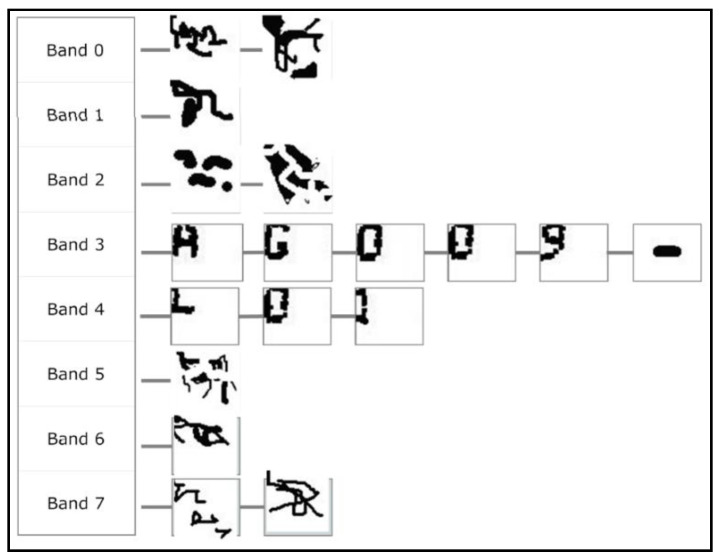
Set of shapes (morphologies) ordered from top to bottom by bands and arranged from left to right. In a typical case some of these morphologies correspond to characters (all morphologies in bands 3 and 4), but others are noise (all morphologies excluding those in bands 3 and 4).

The sequence of processes described in our previous paper [2] (and summarised in the Introduction) produces a set of extracted shapes. Each shape is labelled with the band to which it belongs. It is expected that some of these extracted shapes will indeed correspond to characters, but also that others will be noise. This set will be the starting point for this study. As can be seen in Figure 5, the band label enables the extracted shapes to be ordered from top to bottom. In addition, the shapes have been ordered from left to right. This corresponds to the reading pattern that a human reader would follow. As you will see, the top-to-bottom, left-to-right order is also the “reading” order that MONICOD follows.

Note that this point does not include the absolute position of the characters on the surface of the can, nor the distance between characters other than the line that each character is on and the position it occupies within the line (first, second, third…). The characters will be treated as isolated, individual, and independent morphological entities (represented as templates).

## 4. Comparing Morphologies

MONICOD has a template matching scheme at its core. MONICOD calls these templates “morphologies”. One of the main reasons for having chosen the template matching scheme is that it eliminates the need for, and therefore computational cost of, a complex feature extraction system. This approach makes sense because MONICOD places more emphasis on speed than on the generalisation that may be achieved by classifying a feature vector.

### 4.1. Expected Code

The expiration code can clearly be seen as a composition of characters that are arranged in such a way that each character has individual meaning and is interpreted by its position within the code.

The expected code is constructed with information on the format, the system clock, and the expiration parameter, which depends on the consumer product (e.g., number of months that the product is safe to consume calculated from the packaging date).

As the expected code is considered infallible, if the code extracted from the image does not correspond to it, then the extracted code will be declared incorrect.

It should be noted that each expected code character is represented by the morphologies obtained during the learning process. No “synthetic” morphologies are used.

MONICOD, following the format specified by the operator, generates and internally updates the expected code according to the type of product, calendar and system clock.

### 4.2. Morphologies

**Figure 6:**
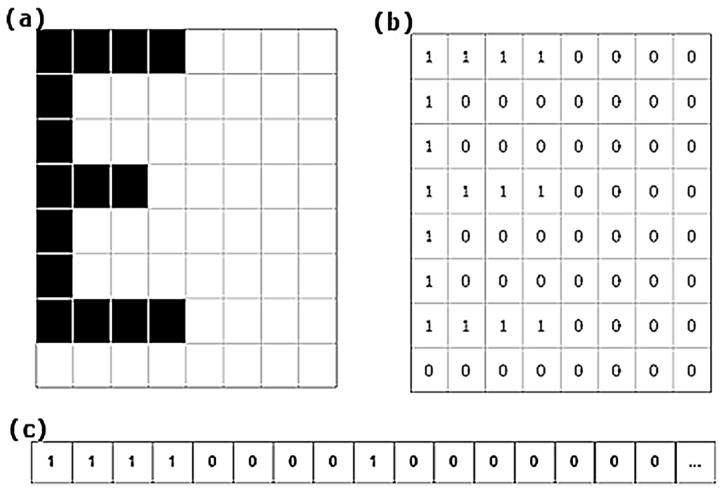
Representation of a morphology in three parts. In (**a**) we have a synthetic example of a binary form. In (**b**) a matrix of zeros and ones that corresponds with (**a**). In (**c**), the matrix (**b**) arranged as a vector which is how MONICOD works internally. This last representation is called a template.

**Figure 7:**
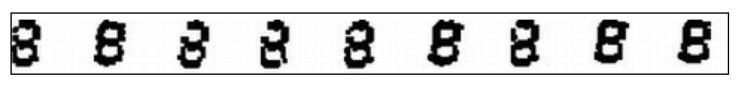
Different morphologies representing the character 8.

A morphology is a discrete rectangular representation of zeros and ones without compression of the m × n dimension. In this representation zero (0) represents non-ink and one (1) ink. The positions of zeros and ones (ink and non-ink) maintain the same relationship as in the shape that they represent. Thus, the morphology is an explicit representation of binary life-size (1:1 scale) shapes (see Figure 6). Later on, we will see that the morphology is the minimum unit of the morphological family database of characters. The reasons for using morphologies include:

- If the shapes are readable and recognisable the associated morphologies will retain those qualities.
- Morphologies are extracted directly from segmentation without any analysis involving new operations such as the creation of a feature vector. There is extensive literature on how to generate a good feature vector [20,21,22,23,24,25,26,27,28], but all these techniques require valuable extra processing.
- Industrial printing standardises. It is expected that printed characters will be similar between cans and produce very similar morphologies (see Figure 7).

### 4.3. Distance between Two Morphologies

The most important action that can be performed with a morphology is to compare it with another morphology and determine the distance or similarity between them; distance and similarity mean in this context the quantitative degree to which the two morphologies are different, or on the contrary, close [29]. MONICOD defines the distance between morphologies on the basis of a simple count of ink and background matches and non-matches between the cells of two morphologies A and B. Table 1 shows a variety of distances, including the one used by MONICOD.

### 4.4. The Morphological Family

**Figure 8:**
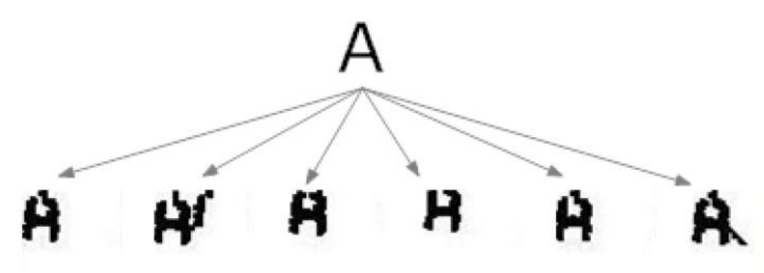
The morphological family of character ‘A’.

The morphological family is a logical grouping of morphologies that responds to the fact that each character is associated with a set or collection of shapes that are equally valid to represent it (see Figure 8). Within the family, two conditions are met:

- Relationship: Every morphology has a greater similarity with any of the morphologies of its own family than with any other morphology belonging to another family.
- No repetition: There are never two identical morphologies within a morphological family.

This concept is due to the distortion produced by printing and image capture. This distortion causes the same character to be associated with multiple morphologies, that is, different distortions of the same shape.

### 4.5. The Morphological Family Database

**Figure 9:**
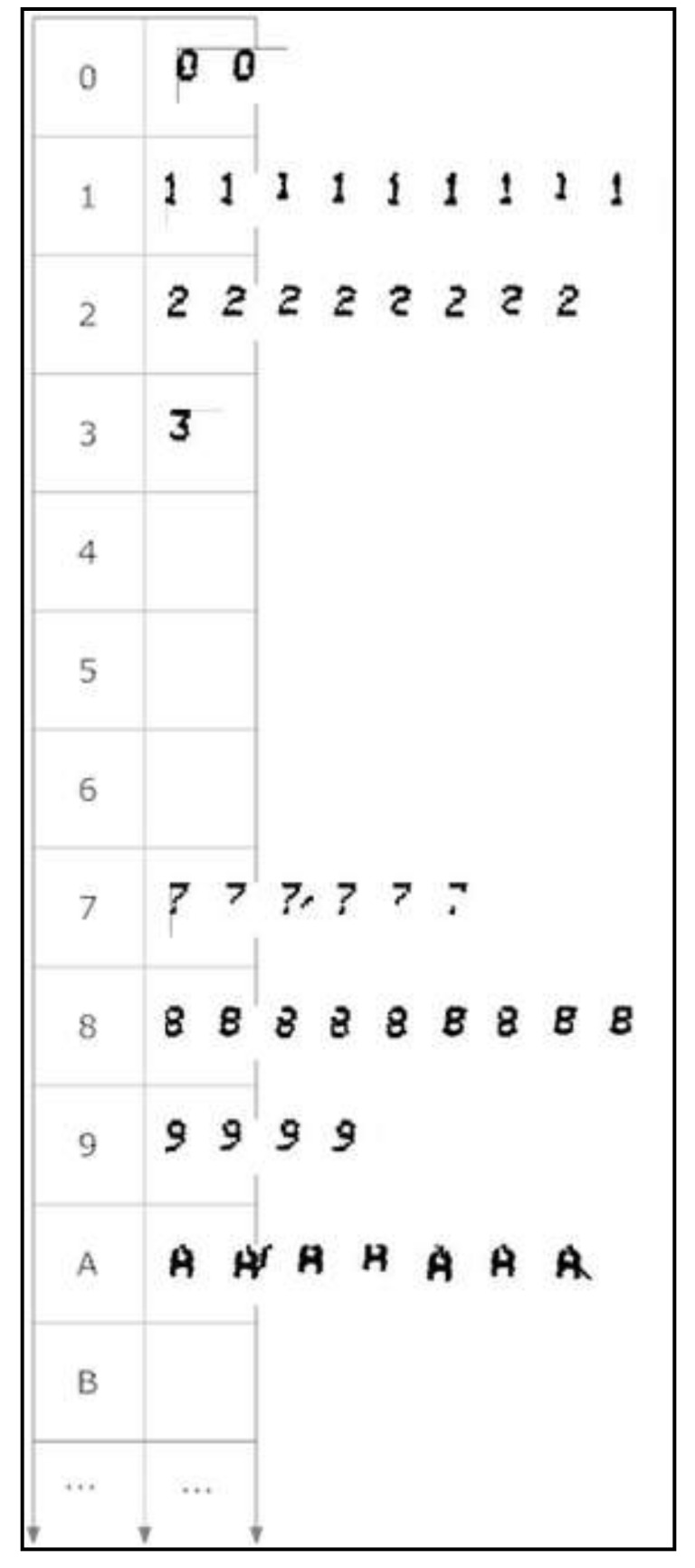
Partial view of the morphological family database. Some characters lack a morphological family because no examples occurred during learning.

The morphological family database (MFD) stores all “learned” morphologies classified into morphological families (see Figure 9). Several operations on the MFD are possible both during validation time and during learning time: addition of a new morphology, deletion of a morphology, repeated consultation of the morphologies of a given morphological family.

### 4.6. Advantages and Disadvantages of Template Matching with MONICOD

The advantages of this template matching scheme are:

- It is intuitive. It can be seen as a systematic matching scheme that assesses the number of ink and non-ink matches between two morphologies.
- It is simple. The only operation involved is the Boolean match and it can be further accelerated with the help of bitwise AND operations.
- It is extremely fast. Template matching involves an intensive but easily paralellisable calculation; following this scheme, the maximum number of operations involved is fixed. Moreover, in a case of validation (like this one) where there is no recognition, we do not need to reach the end of the matching process. In short, the comparison enables us to obtain a partial result as it progresses, so that once a threshold of non-matches is exceeded, we can stop the matching process, and establish that it has failed, as long as we do not need to know the magnitude of the difference.
- It is suitable for this domain. The very repetitive dynamics of can printing hints at the mechanism of template matching: text font, scale, text rotation, etc. These are all fixed properties that should not vary save for the presence of anomalies. The effects of distortion can be attenuated to an extent through the morphological family principle.

**Figure 10:**
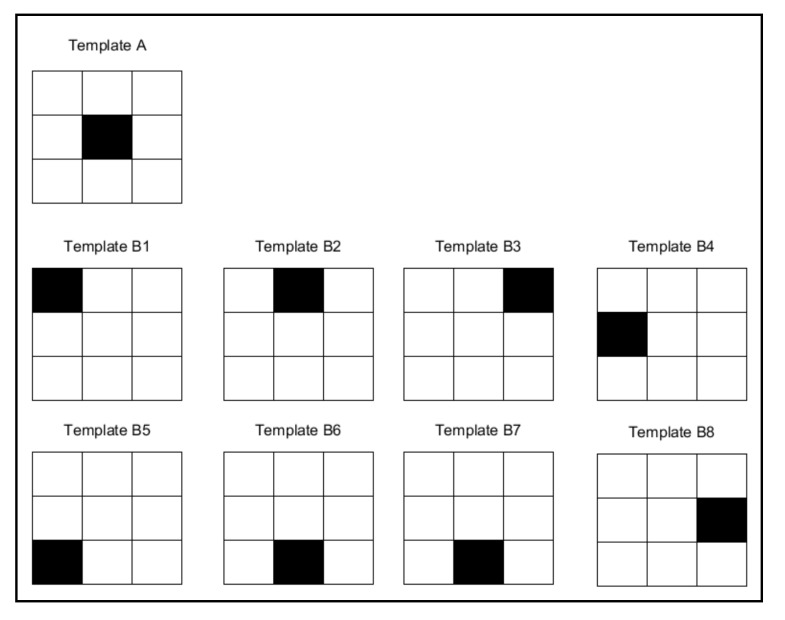
The distance of template “A” from the eight different templates for “B” is the same: the number of ink and non-ink matches is identical.

However, it is not the preferred method for character recognition because:

- It is very sensitive to noise.
- It does not address the relative importance of where matches and discrepancies occur, only their magnitude (the number of times they occur) (see Figure 10). Conversely, a character is distinguished by where exactly the ink and background are located rather than by the quantity involved.
- Low capacity for generalisation. Consequently, it does not support common invariances. It is therefore vulnerable to translation, rotation and scale, among others. Nor does it offer native support for the different equivalent shapes that a character may have. For example, when working with different text fonts.

## 5. Verification

There are two stages in the validation. The first stage, which we call “selection”, is concerned with selecting which expected character we will match with which acquired character, while the second stage is concerned with matching the acquired character and the expected character. We call this verification or matching of morphologies.

### 5.1. Selection

A positive validation requires that the sought-after characters are found on the can. The general rules are:

- The next expected character is not examined until the current one has been found.
- If the acquired character does not correspond to the expected one, the acquired character is assumed to be noise and the process moves on to the next acquired character (but the expected character remains the same).
- Following the matching sequence, matching is performed on a character-by-character basis. This process follows the Western reading pattern in the code to be validated: from left to right and from top to bottom.

In reality, the order of morphology matching is an arbitrary decision as long as we know the reading position (within the code) on which we are reading (in codes there are NO words, nor is there context), but acting in the described way has the following advantage:

- By reading from left to right we have useful information for refining the groupings from the previous stage: we always leave behind complete characters so that, if the current grouping does not correspond to a character, the only alternative is to consider merging it with the grouping on the right.

### 5.2. The Selection Algorithm

**Figure 11:**
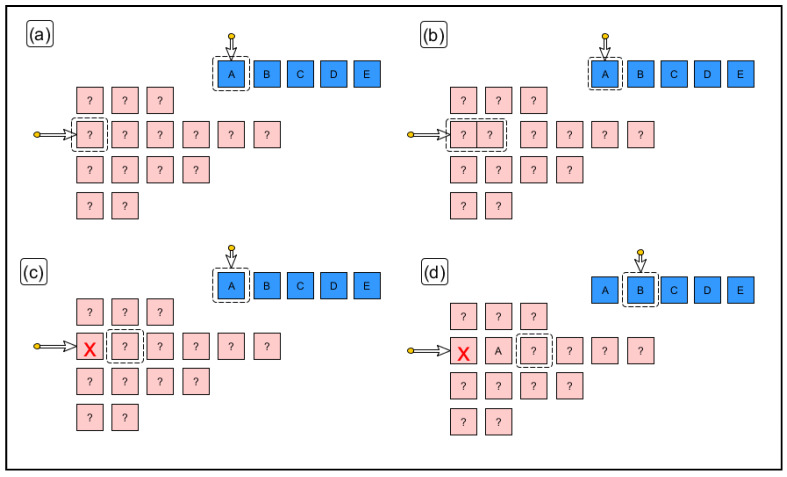
In (**a**) we select the second line of extracted shapes based on the minimum number of characters required by the expected line. The rationale behind the decision is that merging errors are rare. The first shape of the line is also selected. In (**b**) it is shown that if the match with one shape fails, it is merged with the next and matching is reattempted. In (**c**) it is shown that if the step in (**b**) has also failed, that extracted shape is discarded (it must be noise) and we move on to the next one. Finally, in (**d**) a positive match is made. We move on to the next expected character and the next extracted shape.

The following is a comprehensive description of the selection algorithm:

(1) We select an unexamined line (expected code line index) following the top-to-bottom order.
(2) The band index is Positioned on a band suitable for that expected code line following two rules (see Figure 11a):
  (a) The selected band is not above a band that has already been correctly recognised (or previously discarded for that character).
  (b) The selected band has an equal or greater number of shapes than the expected line of code as indicated by the expected line index.
(3) We select the first character of the expected code (expected character index) from the band indicated by the unexamined expected line index following the order from left to right.
(4) The acquired character index points to the first acquired character in the band indicated by the band index.
(5) If there is no character indicated by the expected character index then the expected line is declared to have been fully validated and we return to 1.
(6) If there is no shape indicated then there are missing shapes for this expected line. Another band must be selected, and we return to 2.
(7) We verify the expected character with the acquired character.
  (a) The verification is positive. We move the shape index to the next shape to be verified staying in the same band. We select the next expected character by moving the expected character index to the next position and return to step 5 (see Figure 11d).
  (b) The match is negative. The shape indicated by the shape index is merged—temporarily—with the following shape and the merged shape is compared with the list of morphologies resulting from step 5 (see Figure 11b): First case; If this is a positive match, then it is considered that the two shapes are in fact fragments of the same character. We move the shape index to the following unexamined and unmerged shape. We select the next expected character by moving the expected character index to the next position and return to step 5. Second case; If it is negative, we assume that the source character is noise and move the shape index to the next shape within the current band (see Figure 11c). If the shapes remaining in this band are still sufficient for validation of the expected characters remaining on this line then we return to step 6. Otherwise we return to step 2.

### 5.3. Verification

In binary computing, it is much faster to compare bits than it is to compare integers. Templates, which are vectors of zeros and ones, can be assimilated as bit strings, and thus optimised bitwise operations, such as XOR and AND, can be used to handle them. For example, the scalar product of two bit strings *A·B* provides a bit string that has a number of 1s equal to the number of matches of 1s between the two vectors, i.e., the ink match between two morphologies. With the same procedure, inverting the bit strings $\bar{A}\cdot \bar{B}$, we can evaluate the background matches.

The verification algorithm comprises three distinct phases.

(1) In the first phase, the morphological family corresponding to the expected character is recovered from the MFD. The family must exist, and there must be morphologies within that family. Otherwise we are facing a critical failure in the validation and the operator should be informed immediately.
(2) In the second phase, each of the morphologies of the morphological family is iterated. For each morphology the distance to the acquired “character” is obtained. Section 5.4 Distance Between Morphologies explains how this distance calculation is performed.
(3) The third phase is to select the maximum similarity value (minimum distance) obtained in the previous phase when all the morphologies of the morphological family are compared with the acquired character. If it exceeds a specified threshold, verification is positive. Otherwise it is negative. This result feeds the validation resolution described in Section 5.5 Resolution.

### 5.4. Distance between Morphologies

With MONICOD, we need this critical operation to be performed as quickly as possible. For example, the Euclidean distance would be computationally too costly. MONICOD defines its own distance between morphologies by simply counting the number of matches and non-matches in ink and background between the cells of two morphologies A and B. The counting of ones (or zeros) is a bit-level operation, as is the scalar product between morphologies. The values of the counters enable a simple similarity analysis.

For two morphologies to be comparable they must have the same height and width (m and n). Four independent counters are used. Comparing A and B:

(a) Ink Matches (IM): counts ink matches between A and B.
(b) No Ink Match (NIM): counts background matches between A and B.
(c) Ink Absent (IA): counts non-matches due to ink absent in B that is present in A.
(d) Unexpected Ink (UI): counts non-matches due to ink present in B that is not present in A.

If we exchange operands A and B to B and A, the magnitudes of the counters IA and UI are exchanged, while the counters NIM and IM remain the same. (1) always applies:

NIM + IA + UI + IM = m × n(1)

where IM + IA corresponds to the amount of ink in A, and NIM + UI corresponds to the amount of no ink or background in A.

The matching methodology comprises two sub-phases. The first is limited to counting similarities and differences, cell by cell, between stored morphology and acquired morphology. The position within the morphology is not considered. The second calculates a function, described in (2), parameterised by the count values of the first phase, which provides us with a similarity measurement:

(2) $$1-M(A,B)\;\mathrm{with}\;M(A,B)=\frac{1}{2}\left(\frac{\left|A\cdot B\right|}{\left|A\right|}+\frac{\left|\bar{A}\cdot \bar{B}\right|}{\left|\bar{A}\right|}\right)$$

In expression (2), *A* and *B* correspond to the expected and extracted morphology respectively. $\left|A\right|$ is the number of ones in the vector *A*. *A*·*B* corresponds to a scalar multiplication of two binary matrices (equivalent to a bitwise AND), resulting in a matrix with 1 in the position (i,j) where there is a match of 1 in *A* and *B*, 0 in any other case. Thus, $\left|A\cdot B\right|$ is the number of ones in the scalar product of vectors *A* and *B*. The denominators of the expression are fixed and can be pre-calculated which speeds up calculation of the distance expression. The intuition behind the expression is the average between ratios of ink matches and non-ink matches between two templates.

### 5.5. Resolution

At that point the can is determined to be valid (positive validation) or not (negative validation). This decision is made by means of:

(1) Expected characters verified positively (in position and line) in the acquired can image.
(2) Which characters are important. The configuration of MONICOD by the user provides this information. For example, the numerical characters of time are not important. The year characters are important.

The policy followed by MONICOD is simple: if all the important characters are present in the can on the expected line/order the can is valid. Otherwise it is not.

MONICOD simply updates statistics on total positive and negative validated cans and issues a warning message in the event of a negative validation. The processing of this warning message goes beyond MONICOD and becomes the responsibility of the operator. If the validation is positive it is to be expected that no action will be taken. If the validation is negative, the operator could:

- Ignore the negative validation.
- Remove the affected can manually or mechanically.
- Stop the line to solve a critical problem. In this scenario, system recalibration may be necessary.

## 6. Learning

Within the term “Learning” we include the processes that add or remove morphologies to or from the MFD. With MONICOD, learning occurs in two moments: during learning time and during validation time.

Learning during learning time is intensive: it allows for a considerable transfer of additions and deletions. It is suitable for building the MFD from scratch, although it can just be used to update an existing MFD.

Learning during validation time is adaptive. The aim is to progressively adapt MONICOD to changes that may occur during the day between intensive learning cycles (learning time).

### 6.1. Learning During Learning Time

The learning stage occurs outside the validation cycle as with the calibration stage. As the system is not in operation there are no time constraints and procedures can be applied that would be very costly during validation time. The calibration operation must always precede the learning stage. At this stage, human supervision is required. It consists of two subsystems: automatic and manual.

#### 6.1.1. Automatic Subsystem

This is an automatic template processing subsystem without user intervention. Essentially, the operating principle is similar to the validation process described above with an image capture of a can that is guaranteed to have been correctly printed. The difference is that now the true code obtained can be decomposed and added directly to the MFD as an update of legitimate morphology examples. It will follow an event-based scheme (see Table 2).

**Figure 12:**
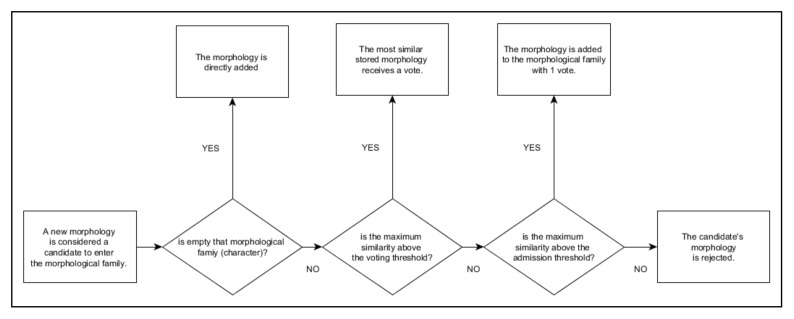
Flow diagram showing how the different tests are applied to decide the fate of a morphology.

The methodology followed by the automatic subsystem is presented below:

(1) First, the conditions for incident-free validation are created. In other words, the printing system prints correctly on the cans, MONICOD has been successfully calibrated and is capable of generating the expected codes.
(2) We start with an empty MFD. Since the MFD is empty, MONICOD cannot validate because it has no morphologies against which to check the input.
(3) Start of the learning cycle for the current can.
(4) The procedure described in [1] is applied until the true code is attained.
(5) For each of the expected characters, the corresponding shape (by position) is extracted from the true code obtained and the MFD is consulted for the associated morphological family. From here, the behaviour will depend on the response of the MFD:
  (a) If there are no morphologies in the MFD for that expected character (morphological family), it is directly added on the basis of the shape extracted from the true code. This is the start morphological family event.
  (b) If morphologies already exist, all the stored morphologies are compared with the shape extracted from the true code to determine which is more similar (maximum similarity). No tied results are possible (because of the way the morphological families are populated). From here there are several possible scenarios (see flow diagram in Figure 12). Scenario 1; If the maximum similarity is above the voting threshold, the selected morphology of the MFD receives a vote. This is the assimilate morphology event (see Table 2). Scenario 2; If the similarity is below the vote threshold, but above the admission threshold, the morphology is added to the morphological family. This is the input morphology event (see Table 2). Scenario 3; If the similarity is below the admission threshold, there has been an anomaly and the candidate’s morphology is rejected. This is the reject morphology event (see Table 2).
(6) Return to 3. The cycle is repeated until a sufficient number of morphologies are available for each morphological family or the supervising user stops the process. Naturally there is a maximum number of morphologies that can be supported. Other stop conditions are possible: stable MFD (no inputs), enough accumulated votes in each morphological family having completed a quota of morphologies for each morphological family, etc.
(7) Once the automatic learning cycle is completed, an automatic purge of morphologies can be carried out based on the number of votes obtained. Morphologies below a given threshold are purged. This is the purge morphology event.

For the time being, the three thresholds—vote threshold, admission threshold and purge threshold—are selected through a trial and error procedure. The vote and admission thresholds are values between 0 and 1.

The vote threshold is always higher than the admission threshold. The closer to 1.0 the more demanding it is. For example, a vote threshold with a value of 1.0 means that a stored morphology will only receive a vote when the incoming morphology is exactly the same.

The values that proved to work best for the vote threshold were in the range of 0.9 to 0.95. The most promising values for the admission threshold appear to be in the range of 0.8 to 0.9.

The purge threshold is indicated as a percentage. For example, a value of 5% means that any morphology that accumulates 5% or less of the total votes for that morphological family will be purged.

#### 6.1.2. Manual Subsystem

The manual template processing subsystem requires user interaction. The aim is to refine the raw results obtained by the automatic subsystem described in Section 6.1.1. The supervisor views the stored morphologies and eliminates them according to his own qualitative criteria. The steps followed by the user are:

(1) View morphology
  (a) The supervisor views the morphology stored in the MFD catalogued by morphological family.
  (b) The user decides whether to delete the morphology or not.
(2) If there are still morphologies to be checked, go back to 1.

**Figure 13:**
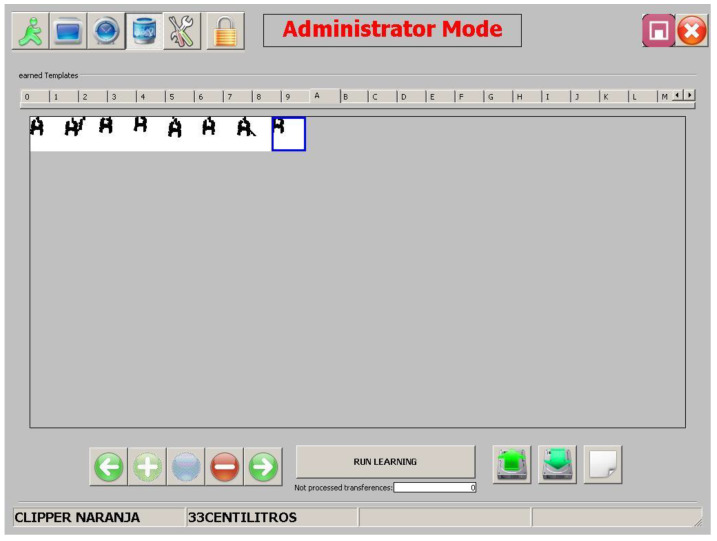
View of the interface of the morphological family database. An operator can select and remove a morphology that is unsuitable or too noisy at the click of a button.

Figure 13 shows the viewer user interface. At present, only the deletion operation is envisaged. Morphologies cannot be moved between morphological families, nor can stored morphologies be edited.

### 6.2. Learning during Validation Time or “on-the-Fly” Learning

This learning stage occurs within the validation cycle. During validation new morphologies are acquired and stored morphologies are eliminated. The aim is to incorporate small changes during operating time. There must be a set of long-term morphologies already stored in the MFD that have been acquired by learning during learning time. No new morphological families are started in this type of learning. The concept of short- and long-term morphology is described below:

- Short-term morphologies are short-lived morphologies that are added and removed at validation time. They bear sufficient similarity to the set of long-term morphologies.
- Long-term morphologies are long-lived morphologies with a minimal degree of readability. Any source character must have a minimum similarity to these morphologies in order to be readable. If it has enough similarity and is sufficiently different from the short-term morphologies currently present, it can be “learned” during validation time and recorded as a short-term morphology. If the number of morphologies for that morphological family is already covered it will replace the least used short-term morphology so far.

The outline of the procedure is as follows:

(1) The validation process is carried out.
(2) One of the following actions is carried out:
  (a) If the code is positively validated and the morphology corresponds 100% to a stored short-term morphology, it is voted for.
  (b) If the code is positively validated and the quota of short-term morphologies is not complete, the morphologies of the code are added to the MFD as short-term morphologies.
  (c) If the code is positively validated and the quota of morphologies is complete, the short-term morphology with the fewest votes is removed and the new morphology is added. Another option is to replace the morphology that has been used the least in recent verifications.
  (d) If the code is NOT positively validated it is ignored for learning purposes.

There is no implementation of this Learning during validation time at the moment.

## 7. Experimental Setup

### 7.1. Description and General Conditions for Obtaining the Input Samples

The conditions for this test are exactly the same as for the previous study [2]. An Intel(R) Xeon(TM) CPU 3.80 GHz, 3.79 GHz 2.00 GB RAM (dual-core) running Windows XP SP3 32-bit was used for the test.

The sample we used was extracted directly from the plant and stored on disk so that the same experiment could be repeated as many times as necessary. A manual check was performed to verify that the cans contained in the sample were correctly labelled.

The stored “video” (9885 frames) corresponded to five independent 10-s recordings. Longer recordings are not possible due to technical issues arising from storing the recording while it is in progress. Each recording was made at 200 frames per second (2000 frames per extraction). The recordings were made in a time frame of approximately 20 min.

They were saved in an uncompressed image format to prevent the introduction of artefacts, colour degradation or loss of frames due to the processing associated with compression (900 kB per frame). The disk space occupied by the sample was 8.48 GB.

The sample was divided into two sets:

- The learning set (composed of 1005 frames). That is, approximately 5 s of non-continuous recording. This set was constructed by merging extracts from each of the five recordings (201 frames per recording). The frames of the set maintained the chronological order of acquisition. The set was manually verified to contain 53 cans. At 200 frames per second, this was a recording of 5.025 s in duration.
- The validation set (composed of 8884 frames). That is, approximately 45 s of non-continuous recording. The frames of the set maintained the chronological order of acquisition. The set was manually verified to contain 465 cans. At 200 frames per second, this was a recording of 44.42 s in duration.

**Figure 14:**
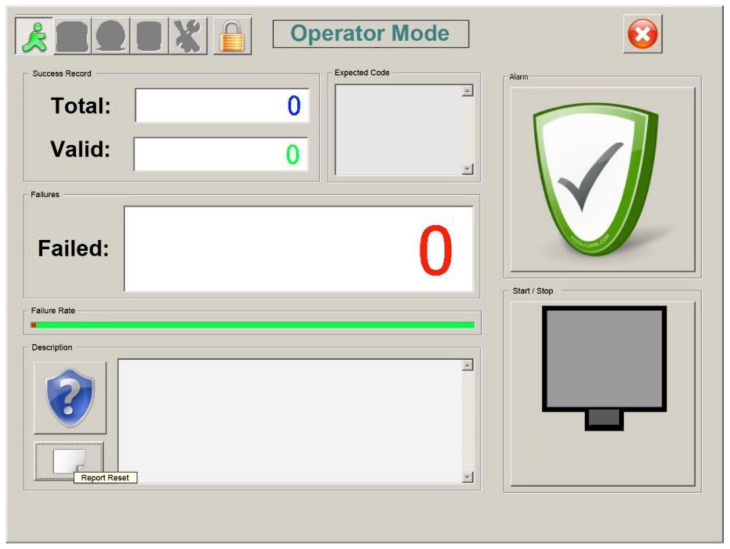
System output interface.

Figure 14 shows the visual output of the system. For practical purposes, the system output comprises three simple counters: total cans, cans that have passed validation, and cans that have failed validation. The ideal scenario is to link this output to some kind of mechanical “ejector” that pushes the can with the wrong expiration code off the conveyor belt. The system should also be able to raise an alarm (light/sound) in the event of too many consecutive ejections, which could be indicative of a chronic printing problem. When alerted by the alarm, the operator would perform a diagnosis and decide what specific action to take (correct it “on the fly”, stop the conveyor belt if there is no alternative, etc.).

### 7.2. Description of the Comparison with Other Classifiers

At the outset, it is important to note that this test uses the MFD and the template matching algorithm or TM, presented in MONICOD, to try to identify which category a morphology belongs to. That is, we use it as a recogniser, and not as a validator. This use is artificial but our expectation is that this way we can evaluate the quality and performance of the algorithms.

A comparison with other popular classifiers was carried out. KNN [30], neural networks MLP with backpropagation [31,32] and RBF [33], and SVM [34,35]. We used three different configurations of the template matching process offered by MONICOD. The difference between the three configurations TM(1), TM(2) and TM(3) is the maximum number of morphologies accepted for a morphological family (8, 4 and 1 respectively). The feature extractor used was Hu’s well-known moments.

It should be taken into account that for this comparison we used morphologies as input (see Figure 12) and not expiration codes. Consequently, we limited ourselves to an assessment of the template matching algorithm and the MFD transfer process, and not other algorithms such as the selection of characters to be compared.

The conditions of the experiment were the same as the previous ones. The MONICOD implementation used was exactly the same as that incorporated in MONICOD. The same applies to the feature extraction code.

The quality criterion was a simple hit/miss count of the classification.

Four different times were taken into consideration for time measurement:

(a) Pre-training time. The time spent on image acquisition (disk reading) and feature extraction for classification.
(b) Pre-classification time. The time spent on image acquisition (disk reading) and feature extraction for classification.
(c) Training time. Time spent on training.
(d) Classification time. Time spent on classification.

The procedure for performing the comparison is as follows:

(1) A basic training set of 8000 samples (templates) is acquired with MONICOD: 1000 samples of each class.
(2) A classification set of 8000 samples (templates) is acquired with MONICOD: 1000 samples of each class.
(3) Repeat the experiment for percentages 0.1, 1, 5, 10, 20, 30, 40, 50, 60, 70, 80, 90 and 100.
  (a) Ten (10) training subsets are constructed by randomly selecting the given percentage from the basic training set. It is always guaranteed that there is the same number of representative samples from each class.
  (b) For each subset (in this case one) and for each classifier: For each subset (in this case one) and for each classifier: Step 1; The features of each template are extracted from the subset and matched to the classifier input. Step 2; The classifier is trained with the subset. Step 3; The features of each template are extracted from the classification set and adapted to the classifier input. Step 4; The complete classification set is classified with the classifier. Step 5; Iteration statistics (time spent in each phase, hits, misses) are recorded. Step 6; The average is calculated with the subset statistics.

## 8. Results and Discussion

### 8.1. Hit Rates and Computational Performance of the Validation Algorithms Presented within MONICOD

**Figure 15:**
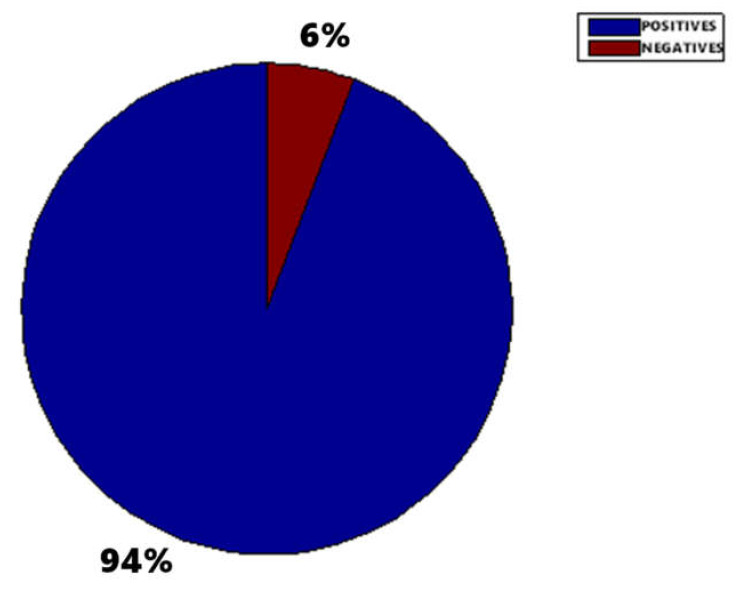
MONICOD validation. All the cans are correct (465) so the 6% (27) of cans that have been negatively validated is erroneous. However, of these 27 wrongly validated cans only one can (0.21%) was caused by the system described in this paper. The rest of the wrongly validated cans were due to errors in the elliptical histogram algorithm, band division and ink fragment grouping (see [1,6,7]).

**Figure 16:**
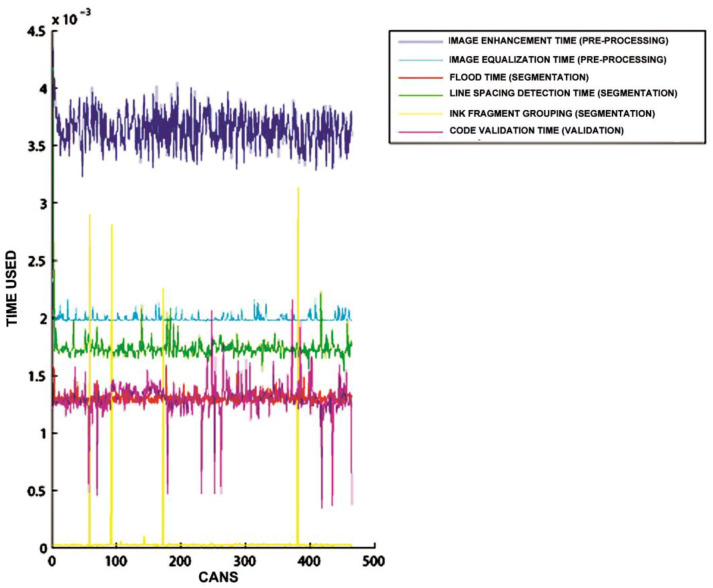
In this graph, the ordinate axis is the time taken, while the abscissa axis represents each of the cans. It can be seen that the validation algorithm has significant oscillations. These oscillations are caused by the variable number of repeated matching attempts with merged fragments and by the variable number of morphologies that are checked until the maximum similarity is obtained.

**Figure 17:**
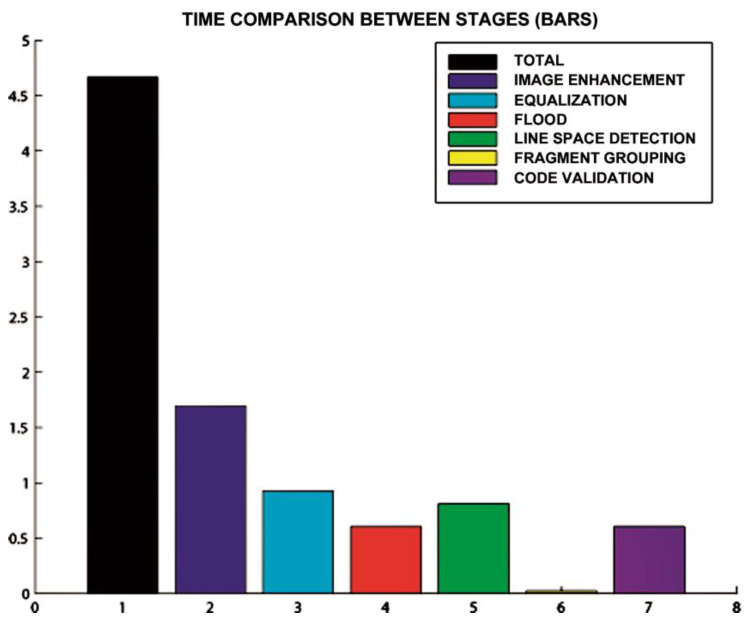
Total time (measured in seconds on the ordinate axis) taken by the algorithms during a recording of 44.42 s. The algorithm described in this paper corresponds to bar 7.

Figure 15, Figure 16 and Figure 17 show the raw results of the experiment involving all the MONICOD steps presented in [2] and in this article. Of the 27 cans that were erroneously validated (5% of the total) only one can be attributed to a failure of the algorithms described in this article. Figure 13; Figure 14 show, on the one hand, that the computational cost involved is low enough to meet the validation target of 200 cans per second and, moreover, makes it clear that this stage is, on average, the second fastest of all the stages comprising MONICOD (only surpassed by the grouping of fragments). This proves that it is by no means a bottleneck for MONICOD. In other words, it is not a high priority for optimisation efforts. The time oscillation shown in Figure 13 between can and can responds to the variable number of mergers performed by the selection algorithm. This is a consequence of imperfect results in the previous stages that the selection algorithm must attenuate. The time spent is also affected by the size of the morphological families for certain characters. The larger the morphological family, the more matches are made between morphologies.

### 8.2. Time and Quality Comparisons between KNN, MLP, RBF, SVM and TM (MONICOD) Classifiers

**Figure 18:**
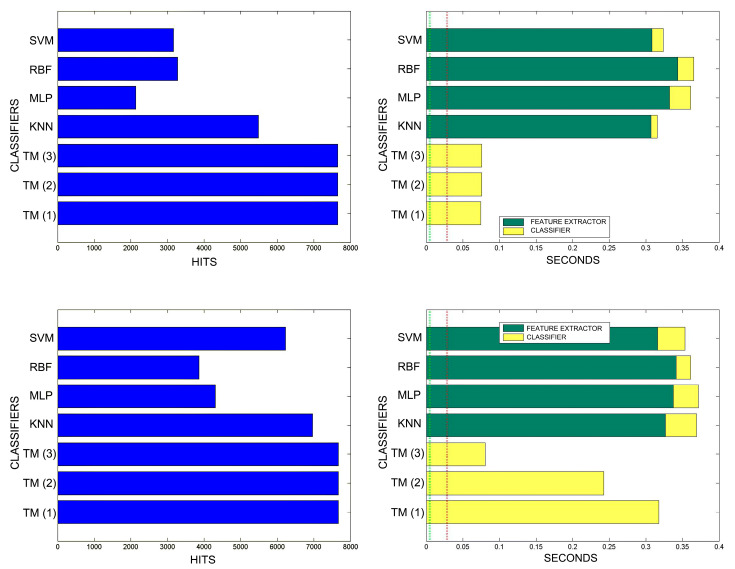

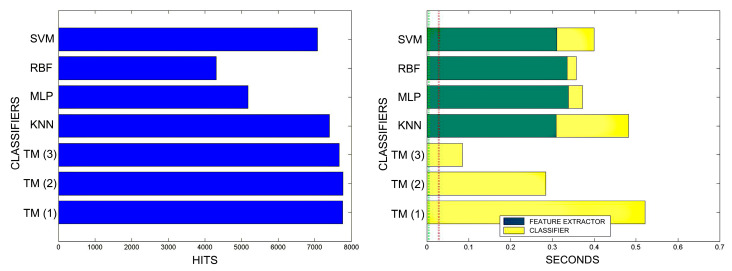
Comparison of successful matches on the left, in blue, and time taken for classification on the right, feature extractor in green, and classification in yellow. In descending order, the training sets represent 0.1%, 1%, 5% of the total training samples.

**Figure 19:**
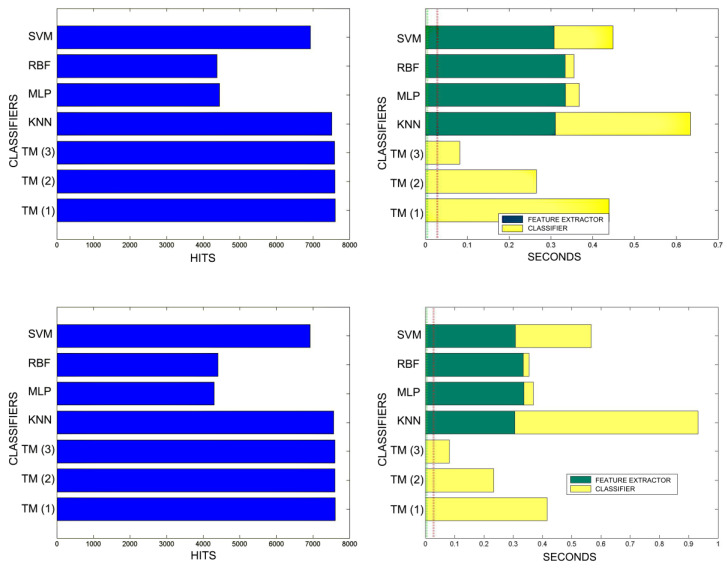
Comparison of successful matches on the left, in blue, and time taken for classification on the right, feature extractor in green, and classification in yellow. In descending order, the training sets represent 10% and 20% of the total training samples.

**Figure 20:**
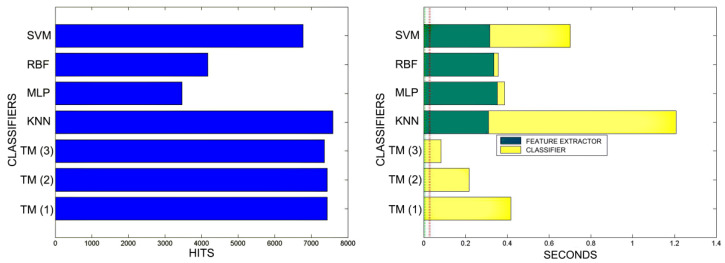

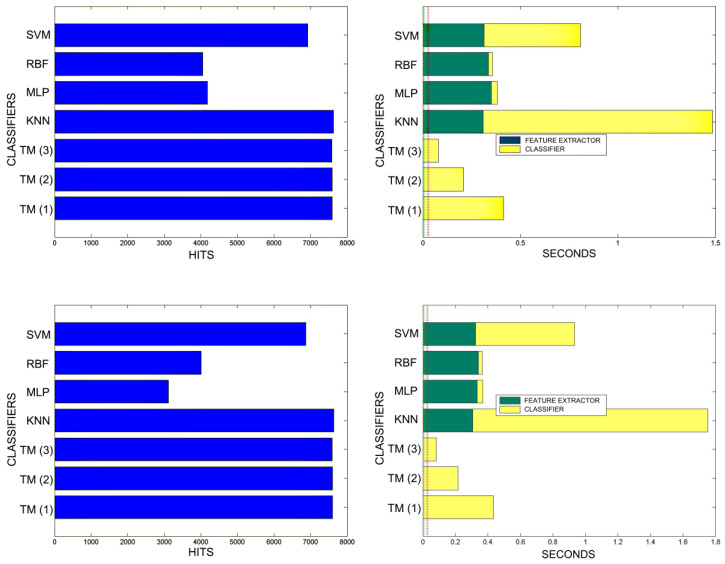
Comparison of successful matches on the left, in blue, and time taken for classification on the right, feature extractor in green, and classification in yellow. In descending order the training sets represent 30%, 40%, 50% of the total training samples.

**Figure 21:**
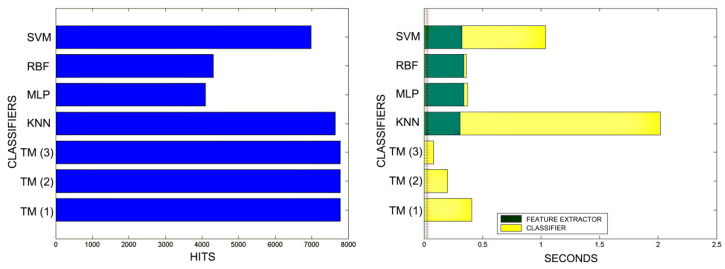

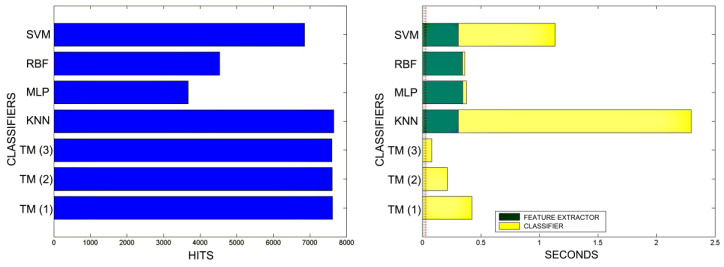
Comparison of successful matches on the left, in blue, and time taken for classification on the right, feature extractor in green, and classification in yellow. In descending order the training sets represent 60% and 70% of the total training samples.

**Figure 22:**
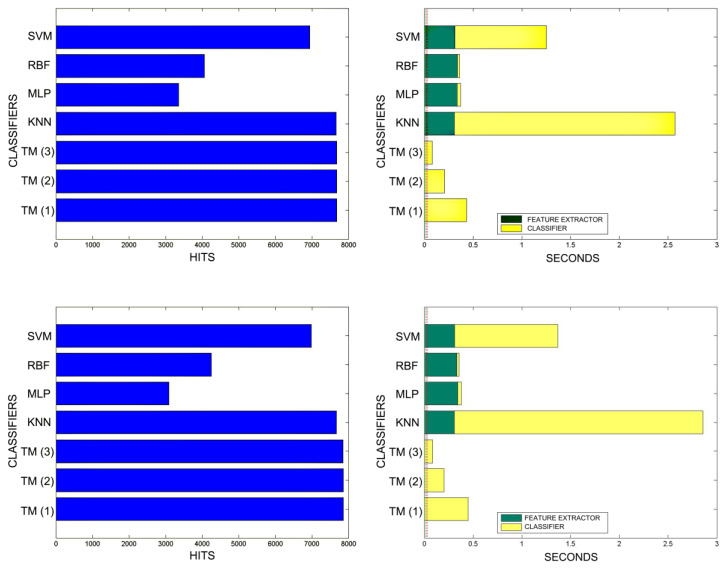

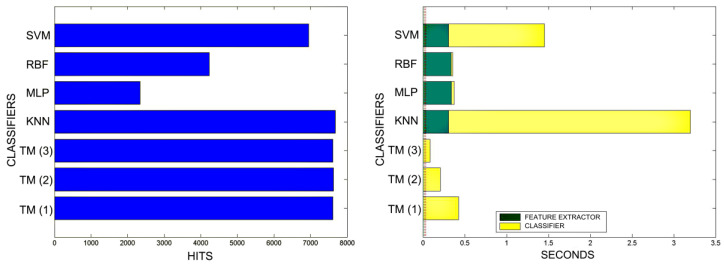
Comparison of successful matches on the left, in blue, and time taken for classification on the right, feature extractor in green, and classification in yellow. In descending order, the training sets represent 80%, 90%, and 100% of the total training samples.

**Figure 23:**
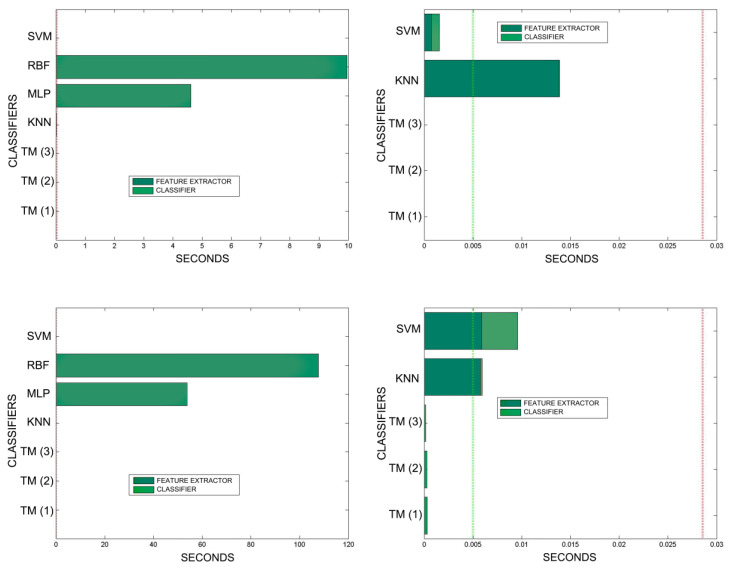

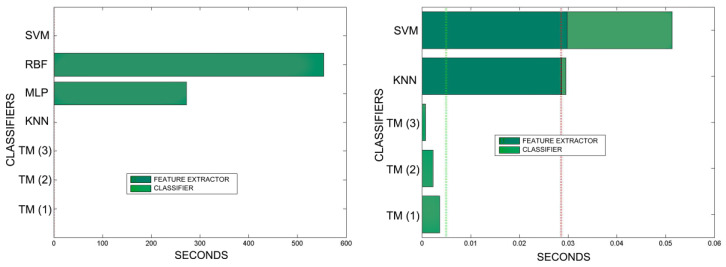
Comparison of training times. 10 averaged training sets of 0.1%, 1%, 5% of total training samples. The graphs on the left show all the classifiers. In the graphs on the right, the RBF and MLP classifiers have been removed in order to better appreciate the training times of the other classifiers. The TM training times are so reduced that they are not appreciated in the graph. This is because TM training consists simply of adding morphologies to the morphological family database.

**Figure 24:**
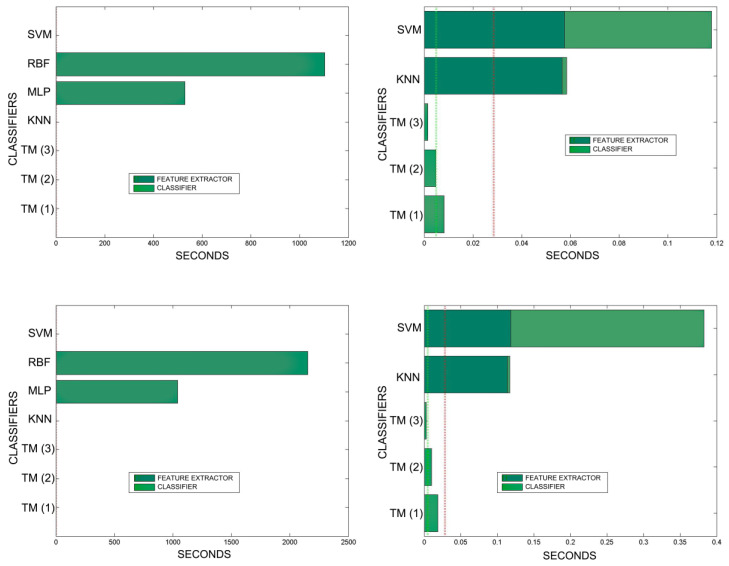
Comparison of training times. 10 averaged training sets of 10% and 20% of total training samples. The graphs on the left show all the classifiers. In the graphs on the right, the RBF and MLP classifiers have been removed in order to better appreciate the training times of the other classifiers. The TM training times are so reduced that they are not appreciated in the graph. This is because TM training consists simply of adding morphologies to the morphological family database.

Figure 18, Figure 19, Figure 20, Figure 21, Figure 22, Figure 23 and Figure 24 show the raw results of the experiment. The main conclusion is that, in our scenario, TM gives a classification quality above 90%, and consistently exhibits the best quality/time ratio. KNN offers comparable efficiency. Other algorithms have inferior qualities, but they can be expected to improve with more thorough training and, in any case, should be offset by the fact that they offer better generalisation (as with neural networks). The generalisation aspect is not taken into account in the experiment because it is a need that is barely touched upon in the domain in which MONICOD operates.

An interesting observation in this respect is that, as might be expected, the graphs show that as the training set becomes larger, the other methods improve their quality of classification until they begin to suffer the effects of over-training. However, TM does not seem to be affected by this factor and does not improve, although it does not worsen either. This result makes sense if one considers how the MONICOD TM proposal works.

Sometimes KNN manages to improve the TM results, but at the cost of a much longer classification time.

It is true that neural networks (MLP and RBF) prove to be much superior in TM classification time (although they are hampered by the need for a feature extractor). However, in the graphs in Figure 18 we see that in training they are extremely slow. This is because MLP and RBF require an iterative process of weight readjustment (in our trial tests, 50,000 iterations were used to train them).

TM learning has the lowest cost, followed by KNN. TM simply includes new templates in the knowledge base where appropriate (the number of comparisons needed is quite low). We are confident that, most probably with a better feature extractor and a more precise configuration, the quality of the classification would improve significantly in the other classifiers, perhaps surpassing TM. But it is unlikely that the time spent on classification will improve.

## 9. Conclusions and Future Work

The final phase of the current implementation of MONICOD—a character validator based on a template matching scheme—has been comprehensively presented.

In the light of the overall results, the algorithm is well within the required speed specifications. The use of high-performance technology based on bit operations and dispensing with a feature extractor are assets that make it very fast compared to alternatives. The filtering of matches also contributes: it only matches when the amount of ink between the morphologies to be compared has been found to be approximately similar. If this is not the case, the similarity is estimated to be zero.

However, the quality offered is not sufficiently satisfactory in terms of false negative validations. Nevertheless, the good results in terms of time are encouraging because they offer room for solutions that will bring about qualitative improvements.

Our comparisons show that with the limited repertoire of characters in an industrial expiration code and under equal conditions, TM (template matching) is faster in classification than KNN and SVM. On the other hand, neural networks (RBF and MLP) can be as fast as or faster than TM in classification, although the quality of classification is lower with the feature extractor used for Hu’s moments.

Under character recognition conditions, TM is the fastest in training time among those compared. Fast enough to deliver the possibility of “on-the-fly” learning. That is, new knowledge can be acquired during classification. This does not seem possible with MPL, RBF and SVM; and difficult to achieve with KNN. TM can make use of the history of past classifications to update the MFD. Hopefully, recent knowledge will prove useful in dealing with effects such as progressive degradation in printing after hours of uninterrupted work. It makes sense because the order in which the codes are presented to the system coincides with the order in which the expiration codes are printed.

Finally, TM is very limited in terms of generalisation, an important index for character recognition. In particular, the character validator is very sensitive to variance. However, the authors believe that in this industrial scenario with such controlled conditions, generalisation is not a priority. In addition, there are other factors to consider:

- There is no variance due to translation. The previous methods keep the character validator ignorant of the position of the character on the can.
- There is no variance in scale. The scale specifications for the printed text are expected to be constant throughout the work day.
- The variance from rotation is an effect of the tilting of the code. However, this effect can be attenuated with simple physical approximation by bringing the camera and printing system closer together. In addition, a catalogue of code rotations could be considered during training.

Among the improvements to be considered we have the possibility of using different well-known distances in addition to the one proposed for MONICOD (see Table 1). In addition, the scheme can be easily internationalised by accepting other alphabets or glyphs. Of course, the number of different characters and how different they are from each other are factors to take into account.

Two learning procedures have also been presented. One supervised, already implemented, which takes place during learning time (the system is not in production) and another, unsupervised. Unsupervised learning has the specific feature that it can function “on the fly”. That is, learning would take place during normal operation time while the system is in production. It should be clarified that this supervised learning has to start from a situation where the system has already received supervised training.

## Figures and Tables

**Table 1:** Distances between binary templates, expressed in terms of logical operators, where |*A*| is the number of ones in vector *A*. *A* corresponds to the expected template. *B* is the template extracted from the image capture. In the case of the MONICOD distance the denominators of the expression are fixed and can be pre-calculated which speeds up the calculation of the distance expression.

Distance	Expression
Hamming	$\left|A⊕B\right|$
Dot product	$\left|A\cdot B\right|$
Jaccard	$1-J(A,B)\;\mathrm{with}\;J(A,B)=\frac{\left|A\cdot B\right|}{\left|A+B\right|}$
Sørensen-Dice	$1-D(A,B)\;\mathrm{with}\;D(A,B)=\frac{2\left|A\cdot B\right|}{{\left|A\right|}^{2}+{\left|B\right|}^{2}}$
Tversky	$T(A,B)=\frac{\left|A\cdot B\right|}{\left|A\cdot B\right|+\alpha \left|A-B\right|+\beta \left|B-A\right|}\;\mathrm{with}\;\alpha ,\beta \geq 0\;\mathrm{and}\;\alpha +\beta =1$
MONICOD	$1-M(A,B)\;\mathrm{with}\;M(A,B)=\frac{1}{2}\left(\frac{\left|A\cdot B\right|}{\left|A\right|}+\frac{\left|\bar{A}\cdot \bar{B}\right|}{\left|\bar{A}\right|}\right)$

**Table 2:** Relationship of events with their meaning for learning during automatic learning time.

Event	Description	Graphic Example
**Start morphological family**	There are no morphologies in this family. Direct addition of the new morphology.	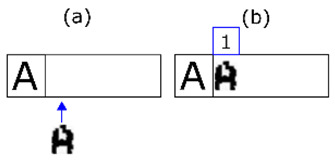
**Assimilate morphology**	The similarity with the most similar morphology in the family is above the voting threshold. Vote for the stored morphology.	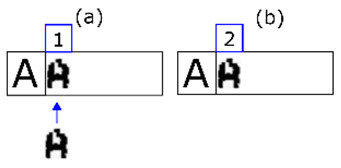
**Input morphology**	The similarity with the most similar morphology in the family is above the admission threshold. The new morphology is added.	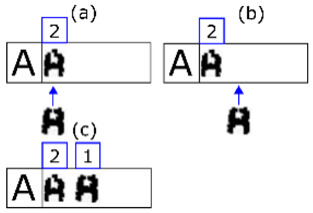
**Reject morphology**	The new morphology is rejected.	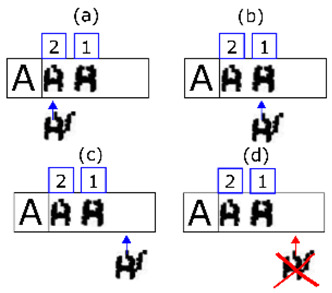
**Purge morphology**	Stored morphologies with number of votes below a given threshold are eliminated.